# Simple and efficient site-directed mutagenesis using two single-primer reactions in parallel to generate mutants for protein structure-function studies

**DOI:** 10.1186/1472-6750-9-61

**Published:** 2009-06-30

**Authors:** Oded Edelheit, Aaron Hanukoglu, Israel Hanukoglu

**Affiliations:** 1 Department of Molecular Biology, Ariel University Center, Ariel, Israel; 2 Department of Pediatrics, Tel-Aviv University, Sackler Medical School, Tel Aviv, Israel; 3 Division of Pediatric Endocrinology, E. Wolfson Medical Center, Holon, Israel

## Abstract

**Background:**

In protein engineering, site-directed mutagenesis methods are used to generate DNA sequences with mutated codons, insertions or deletions. In a widely used method, mutations are generated by PCR using a pair of oligonucleotide primers designed with mismatching nucleotides at the center of the primers. In this method, primer-primer annealing may prevent cloning of mutant cDNAs. To circumvent this problem we developed an alternative procedure that does not use forward-reverse primer pair in the same reaction.

**Results:**

In initial studies we used a double-primer PCR mutagenesis protocol, but sequencing of products showed tandem repeats of primer in cloned DNA. We developed an alternative method that starts with two Single-Primer Reactions IN Parallel using high-fidelity Pwo DNA polymerase. Thus, we call the method with the acronym SPRINP. The SPRINP reactions are then combined, denatured at 95°C, and slowly cooled, promoting random annealing of the parental DNA and the newly synthesized strands. The products are digested with DpnI that digests methylated parental strands, and then transformed into E. coli. Using this method we generated >40 mutants in cDNAs coding for human Epithelial Na^+ ^Channel (ENaC) subunits. The method has been tested for 1–3 bp codon mutation and insertion of a 27 bp epitope tag into cDNAs.

**Conclusion:**

The SPRINP mutagenesis protocol yields mutants reliably and with high fidelity. The use of a single primer in each amplification reaction increases the probability of success of primers relative to previous methods employing a forward and reverse primer pair in the same reaction.

## Background

Site-directed mutagenesis (SDM) methods are used to generate cloned DNAs with modified sequences for examining the importance of specific residues in protein structure and function. SDM represents the primary rational method in protein engineering and for altering enzyme substrate selectivity [1,2].

Currently prevalent methods of SDM employ PCR using oligonucleotide primer pairs that carry the desired mutation. There is a large variety of approaches for SDM using PCR [1,3,4]. One of the widely used methods is QuikChange [5]. This method and its later modifications employ complementary primer pairs in the same PCR reaction. The use of complementary primer pairs may lead to the formation of "primer dimers" by partial annealing of a primer with the second primer in reaction, and formation of tandem repeats of primers, reducing the yield of successful transformants. Primer-primer annealing is especially severe in SDM because the primers used include mismatching nucleotides to generate the desired mutation. To avoid these problems various alternative protocols have been developed [6,7]. Similarly, in our research on the structure-function relationship of human epithelial sodium channel (ENaC) subunits we failed to generate desired clones using a commercial site-directed mutagenesis kit employing double-primers reactions.

We developed a SDM method that includes two PCR reactions run in parallel with each one of the forward and reverse primers. At the end of the PCR, the reactions are combined and after three steps of denaturation, renaturation and DpnI digestion of methylated parental plasmid DNA, the products are directly transformed into host cells.

## Methods

### Plasmids

We cloned the cDNAs encoding for the three subunits of human Epithelial Na^+ ^Channel (α, β and γ ENaC) [8-10] for analyzing structure-function relationships in these proteins. The α, β and γ ENaC cDNA inserts (2013, 1923 and 1950 bp, respectively) were cloned in the expression plasmid pGEM-HJ. The plasmid pGEM-HJ is about 3,000 bp long and includes a T7 promoter used for transcription of cRNA for micro-injection and expression of proteins in Xenopus oocytes. Since the method requires methylated parental plasmid DNA, all plasmids used in these studies were isolated from XL1-Blue cells (Stratagene) that have a dam+ (wild type) genotype encoding Dam methylase.

### Primers

We designed primers for two types of mutagenesis: 1. Mutation of 1–3 nucleotides to change a codon sequence; 2. Insertion of a 27 bp segment for adding a hemagglutinin (HA) peptide epitope tag into cDNA inserts. Table 1 includes seven sets of representative primer sequences we used for site-directed mutagenesis. Forward and reverse primers were designed using guidelines similar to that commonly employed in double-primer PCR reactions:

**Table 1:** Forward (-F) and reverse (-R) primers used for point mutations in α ENaC (a-), and insertion of a 27 nt segment coding for a 9 residue epitope tag in β ENaC (b+) cDNA.

Primer	Sequence*	Length (nt)
a-R333K-F	ctgtccctgatgctgAAGgcagagcagaatgacttc	36
a-R333K-R	gaagtcattctgctctgcCTTcagcatcagggacag	36
a-D338A-F	cgcgcagagcagaatgCcttcattcccctgctg	33
a-D338A-R	cagcaggggaatgaagGcattctgctctgcgcg	33
a-R350A-F	cacagtgactggggccGCggtaatggtgcacggg	34
a-R350A-R	cccgtgcaccattaccGCggccccagtcactgtg	34
a-E358D-F	gtgcacgggcaggatgaTcctgcctttatggatg	34
a-E358D-R	catccataaaggcaggAtcatcctgcccgtgcac	34
a-K474R-F	ctgggctgtttcaccaGgtgccggaagccatgc	33
a-K474R-R	gcatggcttccggcacCtggtgaaacagcccag	33
a-R476K-F	ctgtttcaccaagtgcAAgaagccatgcagcgtg	34
a-R476K-R	cacgctgcatggcttcTTgcacttggtgaaacag	34
b+HA-F	gccattgccaccaggTACCCATACGACGTCCCAGACTACGCTaacctgaacttctcc	57
b+HA-R	ggagaagttcaggttAGCGTAGTCTGGGACGTCGTATGGGTAcctggtggcattggc	57

* Mutated or inserted nucleotides are written in upper case characters.

1. The primer sequences should be complementary to each other.

2. Primer length: 10–20 nt of unmodified sequence on both sides of the mutation.

3. The mutated bases should preferably be in the center of both primers.

4. GC content: 40–70%.

5. Tm: 75–85°C for sequence excluding the mutated site.

6. Primer should start and terminate with at least one G or C.

Primers were ordered from Sigma (Israel) or Syntezz (Israel) and used without further purification.

### SDM using double-primer PCR

In our initial attempts to generate mutants we used the QuikChange^® ^Site-Directed Mutagenesis kit (Stratagene, La Jolla, CA) following the kit protocol without modifications. This protocol employs both forward and reverse primers in the same PCR reaction for 12–18 cycles. The PCR products are denatured, and then reannealed. The non-mutated methylated parental plasmid is digested with DpnI and the remaining plasmids are transformed into E. coli cells.

### SDM using single-primer PCR

**Figure 1:**
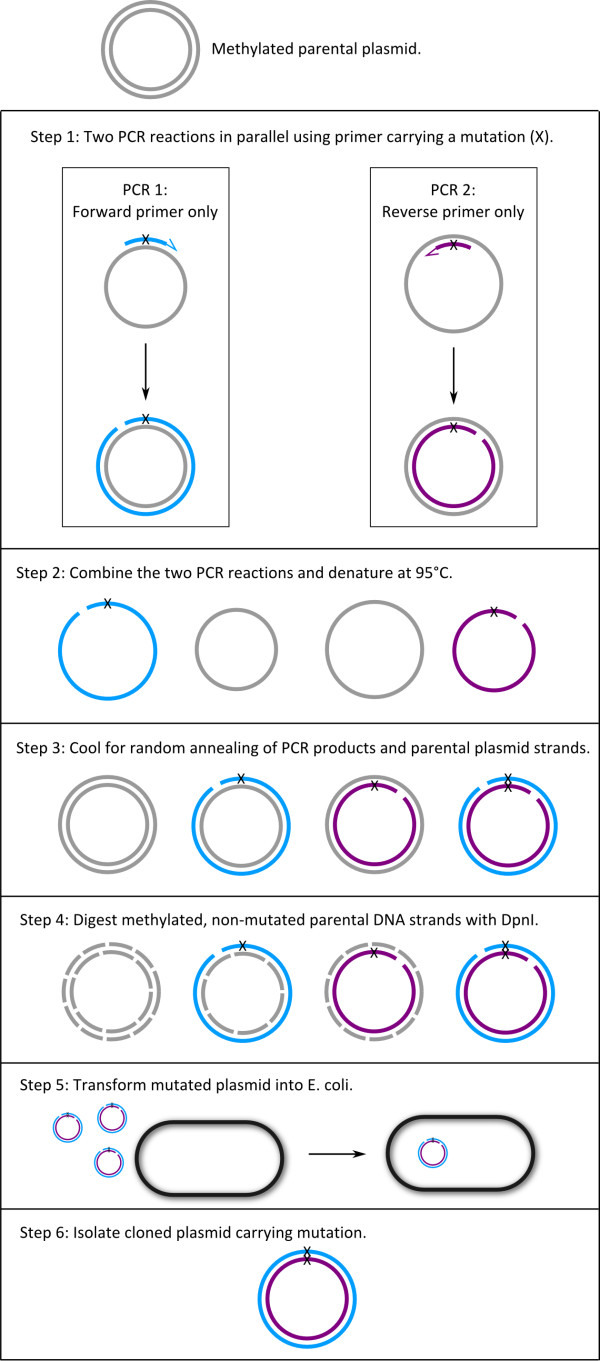
**Flow chart of the single-primer site-directed mutagenesis method**. The parental plasmid is shown in grey color and the two PCR synthesized strands are shown in blue and purple. The letter × marks the position of the mutation.

To avoid the use of forward and reverse primers in the same PCR reaction we developed the following SDM protocol that includes five steps (Figure 1):

1. Amplify the parental plasmid containing cDNA insert in two separate PCR reactions containing either forward or reverse primer.

2. Combine the two single-primer PCR products in one test tube and denature to separate the newly synthesized DNA from the plasmid template DNA.

3. Cool the tube gradually to allow reannealing of the complementary strands.

4. Digest methylated non-mutated DNA (i.e. the parental plasmid) with DpnI.

5. Transform the reannealed plasmids into competent E. coli cells.

For SDM of a cDNA, we carry out two PCR reactions in parallel with the forward and the reverse primers in separate tubes (see Table 2 for reaction components and concentrations). After the initial denaturation step at 94°C for 2 min, PCR was conducted for 30 cycles with denaturation at 94°C for 40 s, primer annealing at 55°C for 40 s and DNA synthesis at 72°C for 60 s for each 1 kb of cDNA + plasmid sequence, e. g. 5 min for a plasmid + cDNA sequence of 5 kb. The DNA polymerase we use is Pwo (Roche, Germany).

**Table 2:** PCR reaction components.

	Reaction 1	Reaction 2
Template plasmid DNA	~500 ng	~500 ng
Forward primer	40 pmol	-
Reverse primer	-	40 pmol
MgCl_2_	0.2 mM	0.2 mM
dNTPs	0.2 mM	0.2 mM
Pwo Master DNA polymerase	1.25 U	1.25 U
Tris buffer, pH 7.5	10 mM	10 mM

Final volume	25 μl	25 μl

After PCR, the two PCR products are combined (giving a total volume of 50 μl) and heated to 95°C to separate the PCR product from the plasmid template. The tube is then slowly cooled to 37°C (Table 3), to promote reannealing of denatured plasmid templates and PCR products (Figure 1). We then add 30 units of DpnI (in 1–3 μl depending on the enzyme concentration; sources of enzyme: Fermentas, NEB and Roche) to the reannealed PCR products (as noted above, in 50 μl) and incubate overnight at 37°C. The restriction enzyme DpnI digests the methylated parental plasmid strands, leaving the newly synthesized strands intact (Figure 1).

**Table 3:** Denaturation and slow cooling conditions to allow reannealing of PCR products.

Step	Temperature (°C)	Time (min)
1	95	5
2	90	1
3	80	1
4	70	0.5
5	60	0.5
6	50	0.5
7	40	0.5
8	37	Holding

Table 4 summarizes the major differences in the initial stages of single-primer and double-primer PCR reactions.

**Table 4:** Comparison of the single-primer and double-primer PCR reactions.

	Single-primer PCR	Double-primer PCR
Number of reactions	2 (1 for each primer)	1
Plasmid template	~500 ng	5–50 ng
Primers	Forward OR reverse (each in a separate tube)	Forward AND reverse together
Primer-primer annealing during PCR	-	+
Number of cycles	30	12–18
Fold amplification of DNA	30	4,096–262,144
Probability of mutation during PCR	Low*	High*
DNA polymerase	Pwo	PfuTurbo

* A function of the fold amplification of DNA.

### Plasmid transformation and isolation

After DpnI digestion, 3 μl of the digest reaction was directly added to 50 μl of competent XL1-Blue cells (Stratagene), incubated for 30 min on ice, heat-shocked at 42°C for 1 min and then transferred to ice for 5 min. After adding 450 μl NZY+ broth, the cells were incubated on a shaker at 37°C for 60 min. Duplicate aliquots of 250 μl of cell suspension was spread on LB plates containing ampicillin (100 μg/ml). After incubating the plates overnight at 37°C, for each transformation we select five colonies at random and grow overnight in 5 ml LB+ampicillin medium at 37°C. The plasmids were isolated using Spin miniprep kit (Qiagen, Germany).

### Sequencing

For the initial check of each isolated plasmid, ~500 ng of the plasmid DNA was taken for sequencing. We have a set of primers that we routinely use for sequencing ENaC cDNAs. For each mutation we selected a sequencing primer about 50–300 bp away from the point of mutation. Sequencing reaction was carried out in a final volume of 20 μl, with the addition of 20 pmol primer, 3 μl sequencing buffer and 2 μl of BigDye^® ^Terminator (ABI). PCR for sequencing was carried out following ABI protocol: Initial denaturation step at 96°C for 1 min followed by 25 cycles of PCR with denaturation at 96°C for 10 s, primer annealing at 55°C for 5 s and extension at 60°C for 4 min. The PCR product was then analyzed using an ABI 310 Genetic Analyzer. If the sequence of the mutated region was found as expected, then the whole cDNA insert was sequenced to ascertain that the cDNA sequence does not carry any other mutation.

The sequence files in ab1 format were analyzed using FinchTV software (Geospiza Inc.). Sequence alignments were done using BioEdit [11].

## Results

### SDM using double-primer PCR

**Figure 2:**
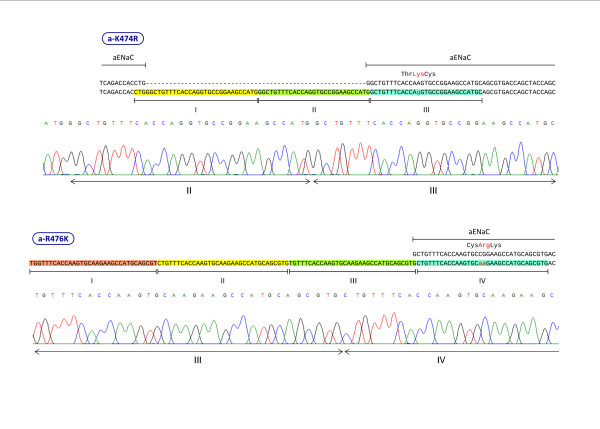
**Sequences of two mutated cDNAs generated with the primer-sets a-K474R and a-R476K using the double-primer PCR method**. The primer sets are listed in Table 1. The mutated residue and the two neighboring residues are shown on top of the DNA sequence. For each primer set, the top DNA sequence is the normal sequence, and the bottom sequence is the sequence of the mutated plasmid. The mutated bases are shown in lower case and red color. Each tandem repeat of the primer is marked with a different background-color and numbered in roman numerals. Note that in the color-marked regions, a few bases are missing from the ends of the primer-copies. These few bases apparently participated in primer-primer annealing. Sequence traces from the ABI Sequencer are shown only for two repeats of the primers to allow visualization of the direct sequencing result. The roman numerals below the traces relate to the roman numerals of the repeats in the respective sequence above the trace.

In initial studies we tried to generate mutants following a widely used double-primer PCR procedure outlined in Methods. In these experiments using primer sets a-R333K and a-E358D (Table 1) we failed to obtain transformants. To identify the source of the problem, we examined the PCR products by gel-electrophoresis. For PCR we used three DNA polymerases from different sources (Pfu from Stratagene, Platinum Pfx DNA Polymerase from Invitrogen and Pwo from Roche). Only PCR with Pwo yielded PCR products. Using two primer sets, a-K474R and a-R476K, we observed PCR products and obtained transformants using the double primer protocol (with Pfu polymerase). We sequenced plasmids from selected colonies using sequencing primers near the mutated site. In both cases primers generated the expected mutation. Yet, the sequences of the plasmids showed tandem repeats of primer sequences in the mutated region (Figure 2). The primer sequences showed overlaps of a few bases, suggesting that these may have originated from primer-primer annealing.

### SDM using single-primer PCR

To overcome the problem of primer-primer annealing observed with the double primer-procedure, we developed an alternative single-primer PCR procedure outlined in Figure 1. We first tested this protocol using the same primer-sets that did not work with the double-primer method. Seeing the success of the protocol we continued to employ it to generate all the mutants needed for our studies.

We start the single-primer PCR protocol with ~500 ng plasmid template that is about 10 times higher than that recommended for the double-primer PCR (Table 4) because, DNA amplification in the single-primer PCR is much lower (Table 4). After each transformation we obtained consistently several hundred colonies. We routinely checked five colonies at random from each transformation by direct sequencing of the plasmid. In 26 transformations that we carried out, the number of plasmids with the designed mutated sequence averaged 3.6 (range: 2–5) out of 5 plasmids from 5 independent colonies. Plasmids that did not have the expected mutated sequence had the original sequence without a mutation. So far, we have not observed tandem repeats of a primer as we observed in the double-primer PCR method (Figure 2).

In 26 SDM experiments, the length of the primers ranged between 31–36 nt. The range for Tm of the primers was 79–92°C and the range for percentage of G+C was 44–76%. These ranges slightly exceed the primer-design guidelines noted in Methods. Since each mutation has to be located at a specific position, we could not always find a suitable primer sequence within the guidelines. Although, we exceeded slightly the guideline range, all the primers worked successfully.

If we define "success" as finding a correctly mutated cDNA among 5 sample colonies examined, then our success rate has been 100% for all single-primer mutation experiments we have carried out. The method has been used in two other laboratories (Prof. Nathan Dascal and Prof. Ilana Lotan, Tel-Aviv University) with a similar success rate of 100%.

### Gel electrophoresis of single-primer PCR products

**Figure 3:**
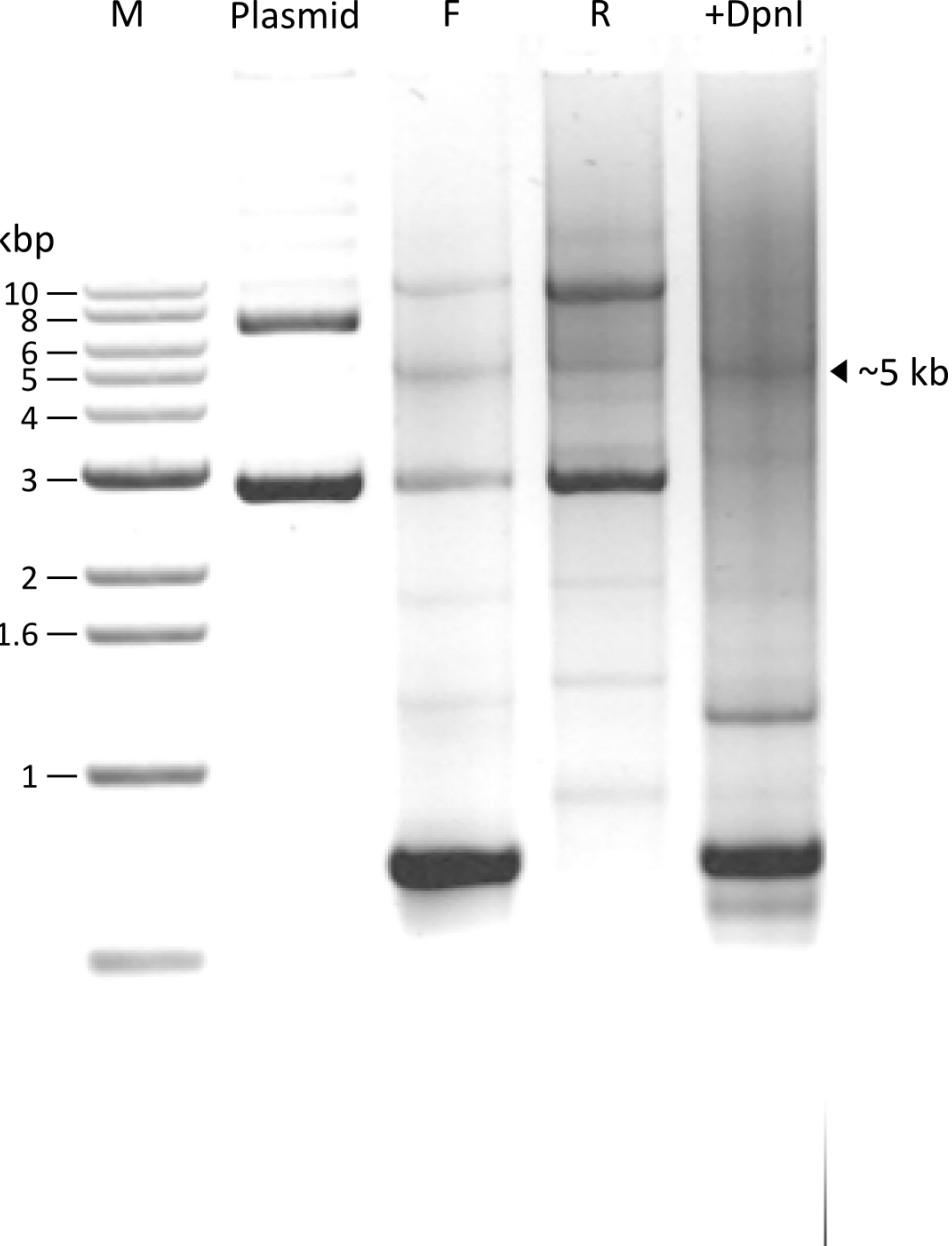
**Separation of DNA products after Single Primer PCR and DpnI digestion reactions**. Plasmid: Parental plasmid alone; F: PCR product with a-D338A Forward primer; R: PCR product with a-D338A Reverse primer; +DpnI: Forward and Reverse primer PCR products combined and digested with DpnI. DNA samples were purified using QIAquick purification kit (Qiagen) and electrophoresed in 1% agarose with Tris-acetate (40 mM Tris, 20 mM sodium acetate, 1 mM EDTA, pH 8.0) as the running buffer. Ethidium bromide stained gel is shown in inverted color (black to white), without any additional digital image editing. The 1 kb DNA ladder marker was from Bioneer (Korea).

The agarose gel in Figure 3 shows the DNA products of one sample at successive steps of our procedure. Plasmid alone, prior to PCR, shows two major bands. After PCR with forward (F) or reverse (R) primers, two major bands can be seen that correspond to parental plasmid DNA. In addition, a new band at the expected size of ~5 kb appears, representing the PCR synthesized linear DNA that includes the cDNA insert and the plasmid. Additional smaller bands represent non-specific PCR products.

Since each PCR reaction is carried out with only one primer, amplification of the DNA is not high (only 30 fold) as compared to the double-primer PCR procedure (Table 4). Therefore, the newly synthesized full size linear plasmid strands do not appear as strong bands on the agarose gel. After DpnI digestion, the two major bands present in the Plasmid lane and in the F and R primer lanes disappear (+DpnI lane), confirming that these two bands represent methylated parental DNAs that were digested by DpnI.

In routine application of our parallel PCR SDM method, we carry out all steps of the method successively without visualizing intermediate products during the whole procedure, i.e., we do not run a gel to examine PCR products. We combine the contents of the two PCR tubes, and proceed with the denaturation and reannealing protocol in Table 3. After this step the DNA is directly taken for transformation into competent E. coli cells.

## Discussion

Site-directed mutagenesis methods are crucial in analyzing structure-function relationships. But, the large number of methods published in the literature attest to the difficulty of executing these methods reliably and efficiently. We observed that widely used double-primer protocol resulted in insertion of multiple copies of primer probably due to primer-primer annealing (Figure 2). The site-directed mutagenesis method we present here circumvents the use of primer pairs in the same PCR. Thus, we call it with the acronym SPRINP (**S**ingle-**P**rimer **R**eactions **IN P**arallel). We have tested the method in our studies on ENaC subunits, and we have also submitted the method for independent testing by two other laboratories as noted in the Results. So far we have observed no instance of multiple primers in the clones we have isolated.

Table 4 provides a list of the differences between the single-primer PCR and the double-primer PCR methods. Probably a most important factor in the success of our method is that we carry out the initial mutagenesis with a single primer in each reaction. In SDM protocols that use forward and reverse primers with complementary sequences, primer-primer annealing can be a significant problem as we observed in our use of double-primer method.

In insertion mutagenesis longer primers with long mismatching segments are used, thus enhancing the probability of non-specific reactions [1]. As noted in Table 1, we used successfully a 57 nt primer (with a 27 nt insertion that did not match the parental strand) in an experiment to introduce an epitope tag into β ENaC cDNA.

In the current study we have introduced mutations and insertions at only one point. Some structure-function studies require multiple-site mutagenesis [12]. We have not examined the use of multiple primers to generate multiple-site mutations. However, the basic principle of the method can be applied for use with multiple primers for multiple-site mutagenesis.

## Conclusion

Using the **S**ingle-**P**rimer **R**eactions **IN P**arallel (SPRINP) mutagenesis protocol that we developed we could generate cDNAs with point mutations and insertions reliably and with high fidelity. The use of a single primer in each amplification reaction increases the probability of successful use of primers relative to previous methods employing a forward and reverse primer pair in the same reaction.

## Authors' contributions

OE developed the new method and carried out the experiments in partial fulfillment of the requirements for a Ph.D. degree at the Sackler Faculty of Medicine, Tel Aviv University, Israel. AH and IH are joint Ph.D. advisers of OE and they were involved in experimental strategies, research funding and are responsible for the writing of the manuscript together with OE. All authors read and approved the final manuscript.
